# Role of microRNAs in premature ovarian insufficiency

**DOI:** 10.1186/s12958-017-0256-3

**Published:** 2017-05-12

**Authors:** Ying Guo, Junyan Sun, Dongmei Lai

**Affiliations:** The International Peace Maternity and Child Health Hospital, School of Medicine, Shanghai Jiaotong University, Shanghai, 200030 China

**Keywords:** Primary ovarian insufficiency, MicroRNAs, Granulosa cells, Ovary

## Abstract

Premature ovarian insufficiency (POI) is a typical disorder of amenorrhea lasting for a minimum of 4 months. The typical characteristics comprised of declined estrogen and raised serum concentrations of follicle-stimulating hormone (FSH) in women <40-year-old, primarily originating from iatrogenic factors, karyotypic abnormalities, and genetic factors. However, the etiology of POI remains unknown in approximately 90% of cases. POI could lead to infertility, osteoporosis, cardiovascular disorder, and cognitive dysfunction. MicroRNAs (miRNAs) are a class of endogenous noncoding RNAs (ncRNAs) that can mediate post-translational silencing of the genes involved in the regulation of proliferation, differentiation, apoptosis, development, tumorigenesis, and hematopoiesis. Recently, the regulatory functions of miRNAs in the development of POI have been the topic of intensive research. The present review addresses the association of miRNAs’ machinery genes (*Dicer*, *Drosha*, and *XPO5*) with POI and the miRNA expression profiles in the plasma of patients with POI. In addition, several specific miRNAs (miR-23a, miR-27a, miR-22-3p, miR-146a, miR-196a, miR-290-295, miR-423, and miR-608) related to POI are also examined in order to highlight the issues that deserve further investigation. A thorough understanding of the exact regulatory roles of miRNAs is imperative to gain novel insights into the etiology of idiopathic POI and offer new research directions in the field.

## Background

Premature ovarian insufficiency (POI) is also referred to as premature ovarian failure (POF) or premature menopause and is characterized by the triad constituting of amenorrhea for at least 4 months, sex steroid deficiency, and monitoring the serum concentrations of follicle-stimulating hormone (FSH) > 40 IU/L, two times at least 1 month apart in women < 40-year-old [1–3]. POI is typical of reduced follicle pool and follicle dysfunction in the ovary and a severe endocrine and reproductive disorder with significant health implications [4]. POI affects the physical and emotional well-being in a woman, resulting in the disorders of the genital tract, poor sexual function, bone and cardiovascular disorder, and cognitive dysfunction [5]. However, the etiology remains undefined in approximately 90% cases [6]. Among the underlying mechanisms, the protein-coding genetic factors are most well-studied. However, the coding exons of the protein-coding genes account for only 1.5% of the genome [7]. During the past few decades, the non-coding portions of the genome have been recognized as epigenetic regulators, crucial for the development, physiology, and disease in the human body. A typical example of the class of most widely studied noncoding regions is microRNAs (miRNAs).

MiRNAs are a class of small noncoding RNAs (ncRNAs), 18–22 nucleotides (nt) in length that can mediate post-translational gene silencing, thereby negatively regulating the target genes [8, 9]. MiRNAs are estimated to regulate the translation of more than 60% of the protein-coding genes and are involved in the regulation of proliferation, differentiation, apoptosis, development, tumorigenesis, and hematopoiesis [10]. The mature miRNAs are generated when the non-coding sequences are processed by RNA polymerase II and the members of the RNase III family, Drosha and Dicer. And exportin 5(*XPO5*) is involved in the following transport process [11]. The role of miRNA in ovaries has been summarized in previous reviews [12–14]. Here, we focus on the recent findings regarding the role of miRNAs associated with POI. An in-depth understanding of the precise functions of miRNAs is imperative that will provide valuable insights into the etiology of idiopathic POI and further the research in the field. Therefore, we surveyed the relative progress and discussed the limitations and persistent issues that necessitate further investigation.

### Diagnosis, etiology and health implications of POI

Menopause is defined as the permanent cessation of menses. The mean (±SD) age for natural menopause is 50 ± 4 years [15]. The diagnosis of POI is confirmed by amenorrhea for a minimum of 4 months, deficiency in sex steroids (estrogen and progesterone), and FSH > 40 IU/L (in normal women FSH is <10 IU/L) in less than 40-year-old women [1–3]. POI affects 1/1000 women by the age of 30 years and 1/100 by 40 years [16, 17].

**Fig. 1:**
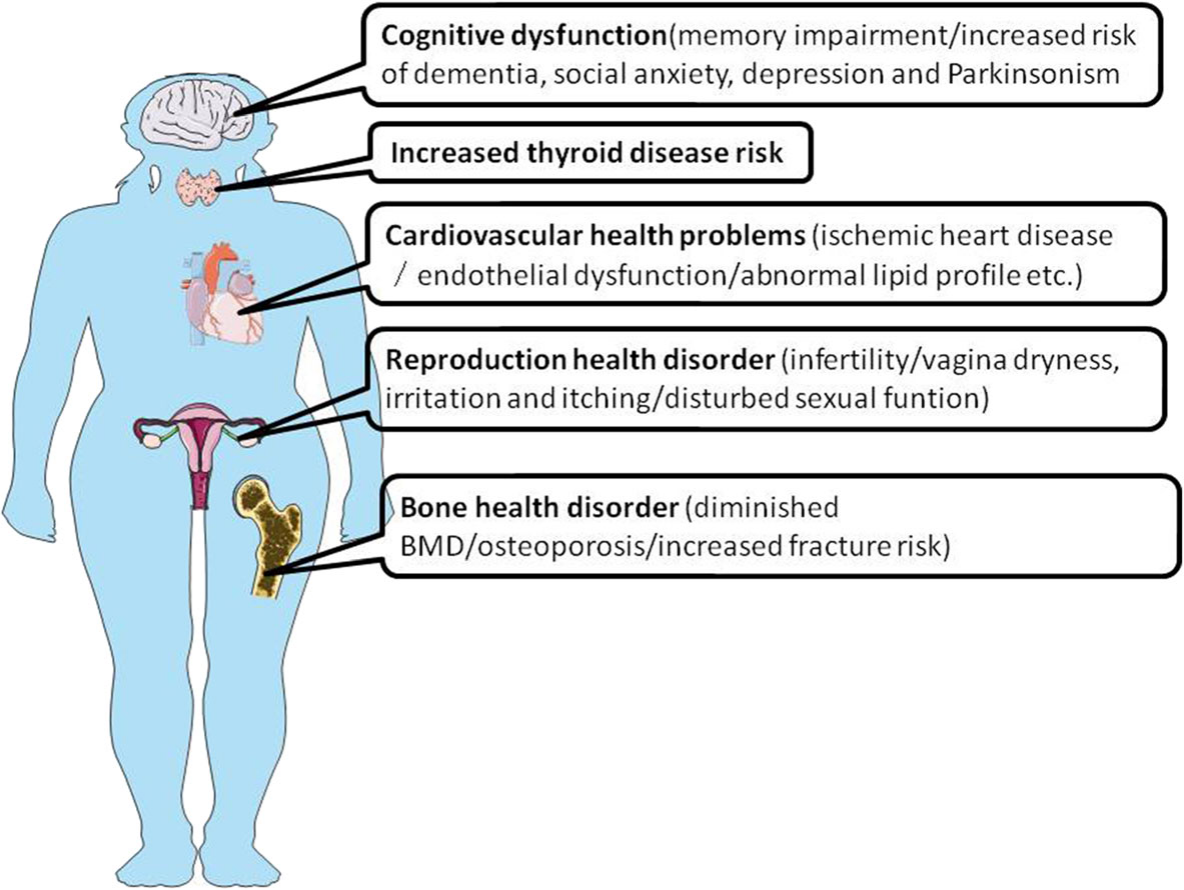
Schematic summary of health implications of POI. POI could lead to cognitive dysfunctions, such as memory impairment, dementia, social anxiety, depression, and Parkinsonism, increase thyroid disease risk, cardiovascular health issues including ischemic heart disease, endothelial dysfunction, and abnormal lipid profile, and cause reproductive and bone health disorders

The ovary is uniquely crucial for reproductive and endocrine functions [1]. The healthy ovarian function is vital for the production of both functional gametes and sex steroids [1]. Several investigations have addressed the health issues in patients with POI. Firstly, women diagnosed with POI suffer from infertility, and the chance of spontaneous conception has been estimated to be 4–10% [18, 19]. Infertility cannot be resolved by in-vitro fertilization (IVF) except by receiving donation cycles of oocyte/embryo [18, 19]. Secondly, women with POI showed worse sexual performance, which could not be resolved by hormonal therapy that was accompanied by increased pain and poorer lubrication than the women with normal gonadal function in the control group [20]. Thirdly, the females with early onset of menopause and premature ovarian failure present a lower value of bone mineral density (BMD) in both femoral neck and lumbar vertebrae [21–26], increasing the bone fragility and risk of fracture than the normal women. Fourthly, the risk of mortality from ischemic heart disease increases up to approximately 80% in the POI group experiencing early menopause as compared to women who underwent late menopause at 49–55 years of age [27]. Finally, the risks of cognitive dysfunction, dementia, social anxiety, depression, Parkinsonism, and impairment of memory are notably increased among women suffering from POI [28–32]. The primary health implications are summarized in Fig. 1.

Although 90% of the POF cases are idiopathic and the etiology in the remaining 10% is largely heterogeneous, major advances have been made in identifying the underlying mechanisms of POI [6]. The causes of POI have been reviewed thoroughly elsewhere including the treatment-related, environmental, metabolic, iatrogenic, autoimmune, and genetic factors [6, 33–35]. With the advancements in the whole genome sequencing, much progress has been made in the analysis of the candidate genes [34]. However, the protein-coding genes are usually under intensive research, whereas the underlying regulatory roles of non-coding sequences in POI are yet to be elucidated.

### MiRNAs

The history of the biological study states that the protein-coding genes have always been the main focus and are well-studied. However, the coding sequences account for only 1.5% of the genome [7]. The term non-coding RNA is employed for RNA that does not encode a protein, but such RNAs also contain information and have functions [36]. Thus, the ncRNAs are gaining increased attention. The ncRNAs are functionally important in development, physiology, and diseases, and are categorized as following: miRNAs, PIWI-interacting RNAs (piRNAs), large intergenic non-coding RNAs (lincRNAs), other long non-coding RNAs (lncRNAs), and circular RNAs (circRNAs). The most widely studied class of ncRNAs is miRNAs, which are small (18–22 nt length) ncRNAs that can mediate the post-translational gene silencing, thereby negatively regulating the target genes.

**Fig. 2:**
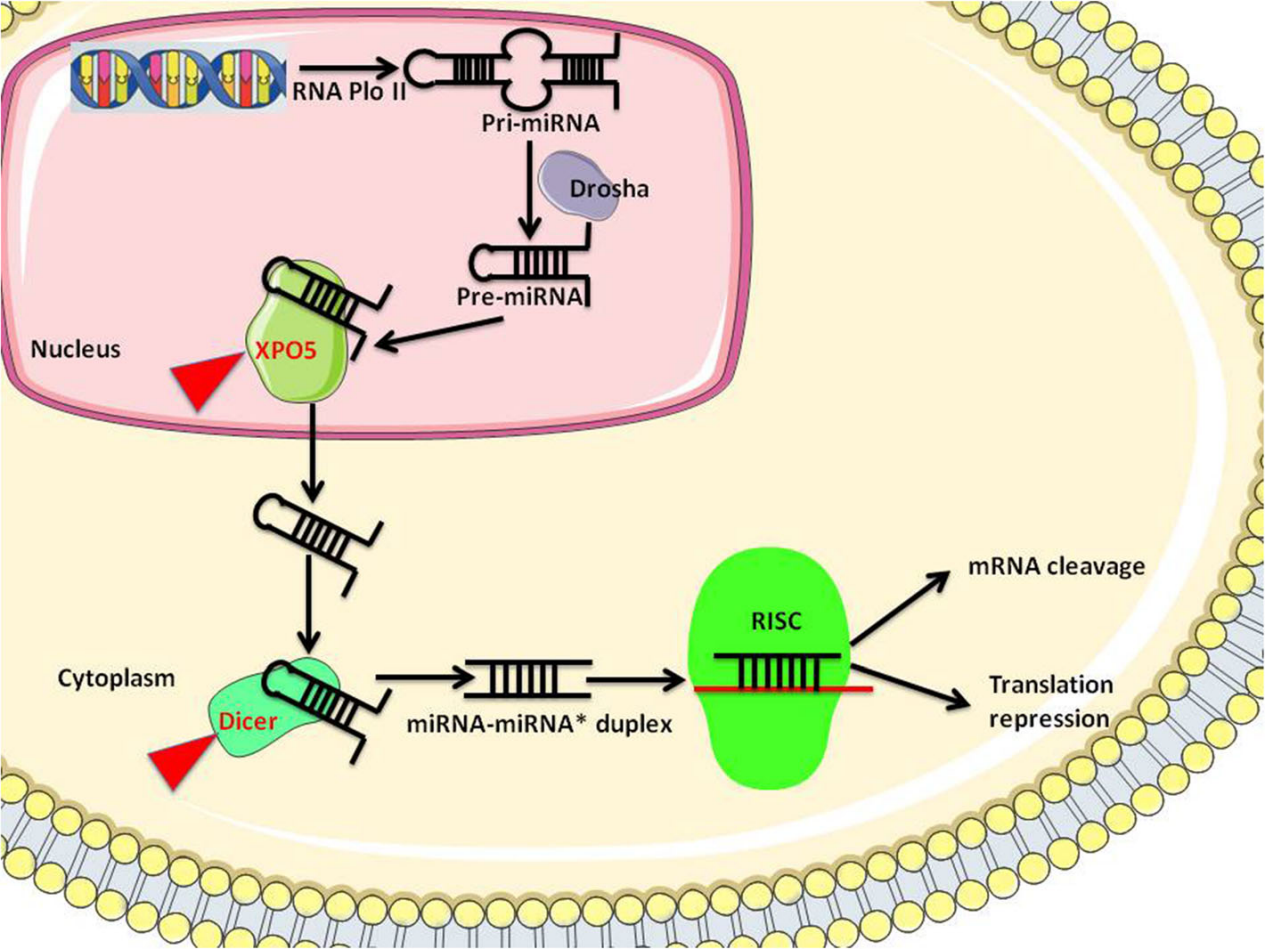
Biosynthesis of miRNA and the machinery molecules associated with POI. MicroRNAs(miRNAs) are transcribed into primary miRNA (pri-miRNA) by RNA polymerase II and are cleaved by RNase III enzyme Drosha complex to generate the precursor miRNAs (pre-miRNAs) in the nucleus. Pre-miRNAs are transported to the cytoplasm by *XPO5*. Pre-miRNAs are further processed by RNase III enzyme, Dicer complex, to form mature miRNAs,which then are incorporated into the RNA-induced silencing complex (RISC), resulting in mRNA degradation or translational repression of target genes. *XPO5* and Dicer were reported to be associated with POI and are marked by *red arrows*

As illustrated in Fig. 2, the biogenesis of miRNAs is a multi-step process [11]. Firstly, the primary precursor molecules (pri-miRNAs), ~500–3000 nt, are transcribed by RNA polymerase II from either independent genes or representative introns of the protein-coding genes. Secondly, the pri-miRNAs are cleaved by the RNase III family member (Drosha) and its cofactor DGCR8 into an ~70 nt pre-miRNA, which then is exported to the cytoplasm by *XPO5*. Thirdly, the pre-miRNAs are processed by Dicer, which is also a member of the RNase III family, assisted by transactivation-responsive (TAR) RNA-binding protein (TRBP) to form a ~20bpmiRNA/miRNA* duplex. One strand of the duplex is a mature miRNA (guide strand) that can be incorporated into a miRNA-induced silencing complex (miRISC), whereas the other strand, termed as the passenger strand, is released and degraded. Most miRNAs target the mRNA by yielding continuous base-pairing of 2–8 nt of miRNA that can be designated as the seed region with complementary sequences in the 3′untranslated regions (UTR) of the target mRNA transcripts. The base-pairing results in either mRNA degradation and deadenylation or translation repression. These miRNAs are ascribed to regulate the translation of > 60% of the protein-coding genes that participate in proliferation, differentiation, apoptosis, development, tumorigenesis, and hematopoiesis [9, 10].

### MiRNAs machinery genes and POI

The global knockout of Dicer and Drosha led to early embryonic lethality in mice (i.e., embryos die at embryonic day 6.5) [37, 38], which emphasizes the developmental role of miRNAs. The loss of function of Dicer or Drosha in prophase I oocytes, developing oocytes, and follicular granulosa cells have demonstrated that miRNAs play critical roles in both the development and functions of the ovary. Selective inactivation of Dicer in prophase I oocytes of the fetal ovary led to compromised folliculogenesis, premature ovarian failure, as well as, infertility in the adult ovary [38]. Dicer1 is enriched in developing oocytes; the conditional knockout of the gene in growing oocytes results in defective spindle organization and chromosome cohesion that in turn, inhibits meiosis I [39], thereby suggesting that Dicer 1 is indispensable for oocyte maturation. However, its function is dispensable for oocyte growth, development, and response to hormonal signals. The inactivation of Dicer1 in the follicular granulosa cells increased the primordial follicle pool endowment, accelerated the early follicle recruitment, and led to increased degeneration of the follicles in the ovaries [40]. Nevertheless, the selective inactivation of Drosha in the oocytes of either the fetal or the developing ovary did not exert any effect on normal folliculogenesis and female fertility in adulthood [38].

Furthermore, the number of studies with respect to the above phenomena is limited in humans. Only one group reported that *XPO5* rs2257082 T variant allele frequently occurs in patients with POI than the controls and this allele may be associated with an increased risk of POI [41]. The molecular machinery associated with POI are illustrated in Fig. 2.

Surprisingly, neither the knockout study of *XPO5* in mice ovary has been conducted nor the single nucleotide polymorphisms (SNPs) of the other genes involved in the molecular machinery have been reported in patients with POI. Hence, loss function of *XPO5* in either oocytes or granulosa cells will shed light on the correlation between miRNA and ovary development. In addition, the analysis of Dicer and Drosha polymorphisms in POF women would also provide further clues about the genetic etiology underlying POF.

### MiRNA expression profiles in the plasma of POI patients

The miRNA expression profiles in the plasma of three idiopathic patients with POI and three control group women with normal cycle were analyzed. Yang et al. identified 10 miRNAs that were significantly up-regulated and 2 miRNAs that were down-regulated in patients with POI [42]. According to another study in Han Chinese patients with POI, a total of 51 differentially expressed miRNAs were identified by miRNA microarray analysis from10 POF and ten control subjects. Of these, 22 miRNAs were significantly up-regulated and 29 were significantly down-regulated in POI group as compared to the control subjects [43], suggesting the essential roles of these miRNAs in POI. Surprisingly, the two groups reported different miRNAs except for miR-23a and let-7c [42, 43]. These different results might at least partially be attributed to the different sample sizes, indicating that the clinical sample size should be expanded in order to obtain persuasive and repeatable results in the future.

### Role of several critical miRNAs in the development of POI

Several miRNAs such as miR-23a, miR-27a, miR-22-3p, miR-146a, miR-196a, miR-290-295, miR-423, and miR-608 have been suggested to be associated with POF. Herein, we addressed the implicated role of these miRNAs in the development of POF.

Both miR-23a and miR-27a are significantly up-regulated in the plasma of women with POI [42, 43], implying the regulatory role of these two miRNAs in the development of POI. Reportedly, miR-23a-27a-24-2 cluster plays critical roles in normal and pathological processes such as cell cycle, proliferation, differentiation, apoptosis, hematopoiesis, and cardiac hypertrophy [44, 45]. MiR-23a induced the apoptosis of granulosa cells by down-regulating the X-linked inhibitor of apoptosis protein (XIAP) with a subsequent increase in caspase-3 cleavage [42]. Both miR-23a and miR-27a mediated the apoptosis of granulosa cells by targeting SMAD5 that in turn, directly activated the Fas ligand (FasL)-Fas pathway in vitro [46]. In addition, Kim et al. found that transfecting a miR-27a mimic sequence into granulosa cells decreased the rate of oocyte maturation in mouse follicles [47]. The insulin growth factor-binding protein 2 (IGFBP-2) produced by granulosa cells regulates the bioavailability of the intra follicular ovarian insulin growth factor (IGF) during follicular development [48]. The IGFBP-2 expression is altered as a result of miRNA-27a transfection; however, the downstream target modulated by this pathway has not yet been explored. Previous studies reported that the expression of IGFBP-2 increased in gonadotrophin-stimulated murine granulosa cells [49], thereby indicating the unbound fraction of IGF as the candidate target. In conclusion, miR-23a and miR-27a are critical for folliculogenesis, putatively mediating the apoptosis of granulosa cells and oocyte maturation via regulating the growth factors during follicular development, and thus, playing a role in the development of POI.

In Chinese patients with idiopathic POF, miR-22-3p was found to be down-regulated and negatively associated with serum FSH [43]. MiR-22-3p has also been reported as one of the most abundant miRNAs in human granulosa cells and is predicted by bioinformatics analysis to negatively regulate the FSH secretion and target estrogen receptor 1 (*ESR1*) and phosphatase and tensin homolog (*PTEN*). *ESR1* and *PTEN* have been considered as candidate genes responsible for POF [34], significantly in folliculogenesis [34]. Moreover, miR-22-3p acts as a suppressor of *ESR1* by directly reducing the mRNA level of the gene in breast cancer cells in vitro [50]. However, the predicted interaction of miR-22-3p with *ESR1* and *PTEN* has not yet been determined in ovaries. The potential roles of miR-22-3p in the pathogenesis of POI and physiological functions remain to be elucidated.

In a Korean study in women with POI [51], the authors demonstrated that the miR-146aC allele coupled with the miR-196a2C allele and the miR-146aG allele coupled with the miR-196a2T allele reduced the risk of POF. However, the miR-146aC allele in combination with the miR-196a2T allele and the miR-146aG allele in combination with the miR-196a2C allele increased the risk of POF [51]. The expression of miR-146a was found to be up-regulated in the plasma and ovarian granulosa cells obtained from idiopathic patients with POI [42, 52]. MiR-146a is expressed in the oocyte during bovine oocyte maturation and preimplantation embryo development, and one of the putative target genes of miR-146a is *Fas* that regulates oocyte apoptosis during folliculogenesis via the caspase8 pathway [53–55]. In addition, miR-146a is also involved in tumor necrosis factor (TNF)-α-dependent regulation of folliculogenesis and atresia by activating a cascade of caspases [56]. Furthermore, miR-146a induced apoptosis in granulosa cells via interleukin-1 receptor-associated kinase (*IRAK1*) and tumor necrosis factor receptor-associated factor 6 (*TRAF6*); these molecules regulated the activity of nuclear factor-kappa B(NF-κB) and inhibitory kappa B α(IκBα), thereby modulating the expression of caspase pathway that could be attenuated by caspase inhibitors [52]. The apoptosis of granulosa cells is pivotal in POI. The understanding of the regulatory mechanisms of miRNAs in apoptosis paves the way for illustrating the pathogenesis of POI. MiR-196a inhibit the expression of newborn ovary homeobox gene (*NOBOX*) during embryogenesis [57]. The mutations in the *NOBOX* gene have been associated with POF [58], suggesting that miR-196a might increase the risk POF by regulating *NOBOX*. Nevertheless, whether NOBOX is regulated by miR-196a in ovary remains to be elucidated. The target genes are possibly influenced by miR-146a and miR-196a; subsequently, the aberrant folliculogenesis occurs. However, the underlying mechanisms are yet to be revealed in future studies.

Recently, Medeiros et al. reported that female miR-290-295-deficient mice are rendered sterile by POF due to the mislocalization of the migrating primordial germ cells (PGCs) [59]. The specific duration of the expression of miR-290-295 explains at least a proportion of idiopathic POF. Strikingly, this cluster is not listed in the miRNAs of adult women with POI.

Another Korean study suggested that breast cancer-related miRNA polymorphisms, including miR-27a A > G, miR-423C > A, and miR-608G > C, are associated with increased idiopathic risk of POI via interaction among the miR-27aG, miR-423A, and miR-608G variants [60]. However, the study should be further substantiated owing to the small sample size and low statistical power.

Amniotic fluid stem cells (AFSCs)-derived exosomal miR-10a contributes significantly to prevent POF caused by chemotherapy in mice [61]. Therefore, the study provides new insights into POF, thereby advancing the therapeutics. In addition, the work suggest that loss function of miR-10a in ovary may help to illuminate the connection between miR-10a and POF in the future.

## Conclusions

Dicer is vital for folliculogenesis, maturation of oocytes, and follicle recruitment. *XPO5* polymorphism is correlated with the increased risk of POI. In addition, differentially expressed miRNAs have been revealed between patients with POI and control group women which are involved in granulosa cells apoptosis, oocyte maturation, oocyte apoptosis, recruitment of primordial follicle, and localization of migrating PGCs (Table 1). However, several issues are yet to be resolved. Firstly, the larger sample size is advised in further studies to draw a substantial conclusion. Secondly, some miRNAs are determined to have modulatory functions in the ovary; whether complete dysfunction of single miRNA will lead to POI is yet unknown. The loss of function of single miRNA in ovary via novel genetic strategy is a promising approach to discover additional functions of miRNA in POF. Thirdly, most of the regulatory and functional networks of miRNAs remain unknown, and the software-predicted targets of specific miRNAs necessitate further verification, which will facilitate the understanding of POI and developing new approaches for the treatment of POI.

**Table 1:** miRNAs associated with POI

miRNA associated with POI	Function	Reference
miR-23a	apoptosis of granulosa cells	[45, 62]
miR-27a	apoptosis of granulosa cells oocyte maturation	[5, 45, 46]
miR-22-3p	unknown	[42]
miR-146a	oocyte apoptosis apoptosis of granulosa cells	[50, 51]
miR-196a	unknown	[50]
miR-290-295	location of the migrating PGCs	[58]
miR-423	unknown	[63]
miR-608	unknown	[63]
